# Anti-Neuroinflammatory Effects of a Novel Bile Acid Derivative

**DOI:** 10.3390/ijms25137136

**Published:** 2024-06-28

**Authors:** Srđan Bjedov, Goran Stegnjaić, Suzana Stanisavljević, Milica Lazarević, Ivan Pilipović, Marija Sakač, Đorđe Miljković

**Affiliations:** 1 Department of Chemistry, Biochemistry, and Environmental Protection, Faculty of Sciences, University of Novi Sad, Trg Dositeja Obradovića 3, 21000 Novi Sad, Serbia; srdjan.bjedov@dh.uns.ac.rs (S.B.); marija.sakac@dh.uns.ac.rs (M.S.); 2 Department of Immunology, Institute for Biological Research “Siniša Stanković”—National Institute of the Republic of Serbia, University of Belgrade, Bulevar Despota Stefana 142, 11108 Belgrade, Serbia; goran.stegnjaic@ibiss.bg.ac.rs (G.S.); ssuzana@ibiss.bg.ac.rs (S.S.); milica.lazarevic@ibiss.bg.ac.rs (M.L.); ivan.pilipovic@ibiss.bg.ac.rs (I.P.)

**Keywords:** Nrf2, Nrf2 activator, T cells, microglia, myeloid cells, cytokine

## Abstract

In the search for novel potent immunomodulatory nuclear factor-erythroid 2 related factor 2 (Nrf2) activators, a derivative of cholic bile acid, SB140, was synthesized. The synthesis of SB140 aimed to increase the electrophilic functionality of the compound, enhancing its ability to activate Nrf2. Effects of SB140 on microglial cells, myeloid-derived cells (MDC), and T cells were explored in the context of (central nervous system) CNS autoimmunity. SB140 potently activated Nrf2 signaling in MDC and microglia. It was efficient in reducing the ability of microglial cells to produce inflammatory nitric oxide, interleukin (IL)-6, and tumor necrosis factor (TNF). Also, SB140 reduced the proliferation of encephalitogenic T cells and the production of their effector cytokines: IL-17 and interferon (IFN)-γ. On the contrary, the effects of SB140 on anti-inflammatory IL-10 production in microglial and encephalitogenic T cells were limited or absent. These results show that SB140 is a potent Nrf2 activator, as well as an immunomodulatory compound. Thus, further research on the application of SB140 in the treatment of neuroinflammatory diseases is warranted. Animal models of multiple sclerosis and other inflammatory neurological disorders will be a suitable choice for such studies.

## 1. Introduction

An increase in the incidence of autoimmune and chronic inflammatory diseases in modern societies urges the development of novel immunomodulatory therapies. Glucocorticoid-based drugs are highly efficient yet with considerable side effects. In an attempt to create novel compounds that would work through glucocorticoid receptors, we have recently synthesized a number of bile acid derivatives [1]. Some of these compounds were also tailored to be potential activators of nuclear factor-erythroid 2 related factor 2 (Nrf2), i.e., to react with Kelch-like ECH-associated protein1 (Keap1). Namely, it is necessary to release Nrf2 from Keap1 in order to enable Nrf2 nuclear translocation and consequent activation of the antioxidant response element (ARE) and expression of phase 2 enzymes [2]. Electrophiles have been shown to be especially active in removing Keap1 from the complex with Nrf2 [2]. One of the drugs that is used for the therapy of multiple sclerosis and psoriasis is dimethyl fumarate, an electrophilic compound with profound Nrf2-activating properties [3]. Thus, our strategy in designing bile acid derivatives was to increase their electrophilic properties. SB140 is one of these compounds, and its Nrf2-activating and immunomodulatory properties were explored in this study.

It is widely accepted that the immune pathogenesis of multiple sclerosis starts with activation of central nervous system (CNS)-reactive T cells, which are differentiated towards interferon (IFN)-γ-producing T helper (Th)1 and/or interleukin (IL)-17-producing Th17 cells [4]. The activation and differentiation of T cells are dependent on antigen-presenting cells, dendritic cells in particular [5]. Autoreactive T cells orchestrate autoimmune response directed towards structures of the CNS and lead to full-blown inflammation, which involves the activity of other relevant effector cells, such as microglia and macrophages [4,6,7]. These cells are also involved in the pathogenesis of other inflammatory CNS diseases, including prominent neurodegenerative disorders such as Parkinson’s and Alzheimer’s disease [8,9,10,11].

Thus, the effects of SB140 on dominant neuroinflammatory cells: microglial cells, myeloid-derived cells (MDC), i.e., macrophages and dendritic cells, and T cells were examined in this study. Also, the ability of SB140 to activate Nrf2 was determined in microglial cells and MDC.

## 2. Results

### 2.1. Design of SB140

**Figure 1:**
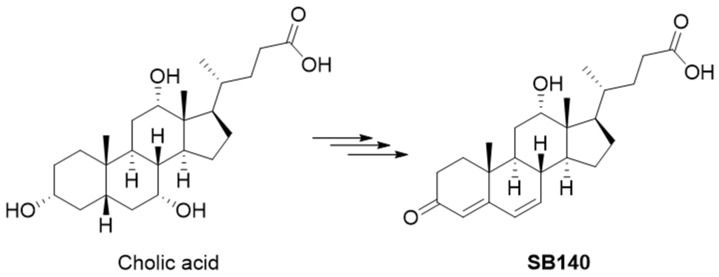
Synthesis of SB140 starting from the cholic acid.

SB140 was designed to include an electrophilic dienone moiety in the steroidal A and B rings while preserving the carboxylic group from the starting bile acid. This carboxylic group can be further modified to improve ADMET properties without compromising the dienone pharmacophore in the steroidal skeleton. The design of SB140 was based on the hypothesis that flattening the classical bile acid stereochemical properties, specifically the cis A/B ring junction, would allow the compound to more easily access cysteine residues crucial for the Keap1/Nrf2 interaction. SB140 was synthesized through a few-step reaction sequence starting from cholic acid (Figure 1).

### 2.2. SB140 Exerts Immunomodulatory Effects on Microglial Cells but Not on MDC

**Figure 2:**
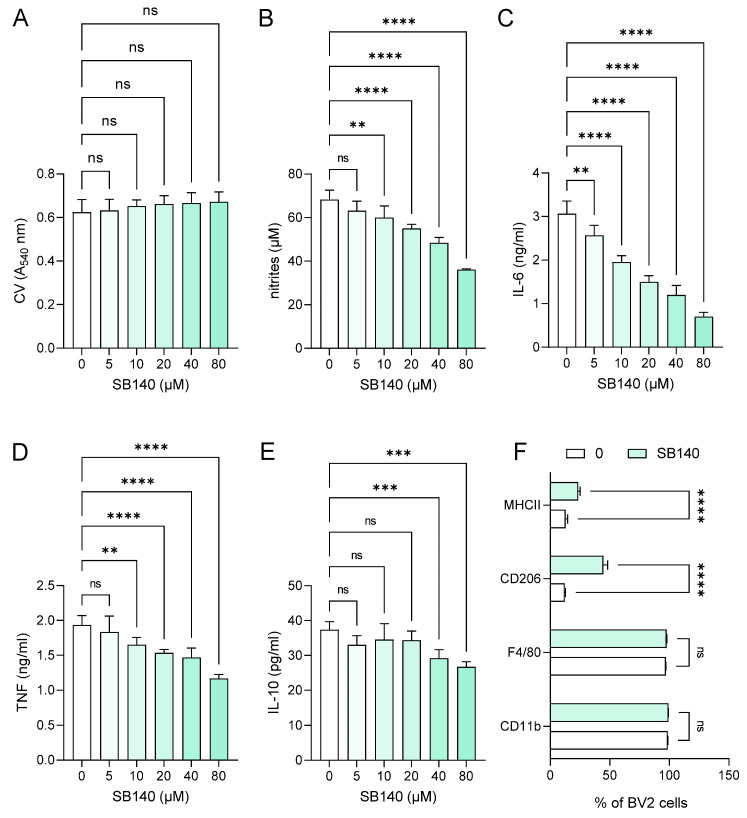
Effects of SB140 on microglial cells. BV2 cells were stimulated with interferon (IFN)-γ (10 ng/mL) and lipopolysaccharide (LPS) (10 ng/mL) in the absence (0) or presence of various concentrations of SB140 for 24 h. Subsequently, cell viability was determined by crystal violet (CV) assay (**A**), nitrite accumulation in cell culture supernatants was measured by Griess reaction (**B**), levels of interleukin (IL)-6, tumor necrosis factor (TNF), and IL-10 in cell culture supernatants were determined by ELISA (**C**–**E**), while the percentage of BV2 cells expressing CD11b, F4/80, CD206, or MHC class II molecules was determined by flow cytometry (**F**). Data from four experiments (**A**–**E**) or from five samples (**F**) are presented as mean + SD. ** *p* < 0.01; *** *p* < 0.001; **** *p* < 0.0001; ns—no statistical difference; repeated measures one-way ANOVA followed by Dunnett’s multiple comparisons test (**A**–**E**) or *t*-test (**F**).

**Figure 3:**
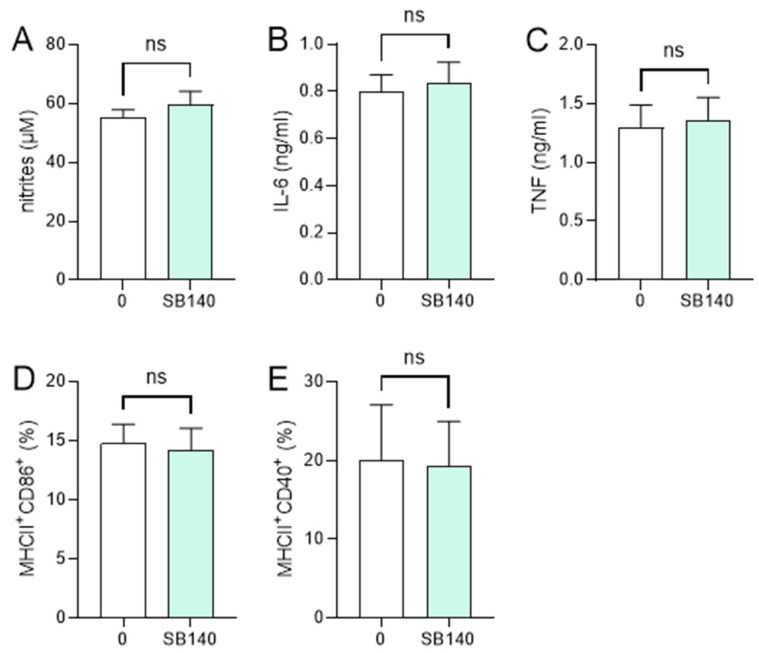
Effects of SB140 on myeloid-derived cells (MDC). MDC were stimulated with LPS (100 ng/mL) in the absence (0) or presence of SB140 (10 μM) for 24 h. Subsequently, nitrite accumulation in cell culture supernatants was measured by Griess reaction (**A**), levels of IL-6 and TNF in cell culture supernatants were determined by ELISA (**B**,**C**), while the percentage of cells expressing MHC class II and CD86 or CD40 was determined by flow cytometry (**D**,**E**). Data from four experiments are presented as mean + SD. ns—no statistical difference; unpaired *t*-test.

In order to explore the effects of SB140 on microglial cells, BV2 cells were stimulated with IFN-γ+ lipopolysaccharide (LPS) and treated with various concentrations of SB140. While the compound did not affect BV2 cell viability (Figure 2A), it potently and dose-dependently reduced their ability to produce nitric oxide (NO) (Figure 2B), IL-6 (Figure 2C), and tumor necrosis factor (TNF) (Figure 2D). Although there was a statistically significant influence of SB140 on anti-inflammatory IL-10 production in BV2 cells at higher concentrations (Figure 2E), it was limited and much less than its effect on proinflammatory cytokines. Further, SB140 did not affect the proportion of microglial cells expressing CD11b and F4/80, but it markedly increased the percentage of BV2 cells expressing CD206 and, to a lesser extent, the proportion of those expressing MHC class II molecules (Figure 2F). Thus, SB140 potently suppressed the inflammatory properties of microglial cells. Effects of SB140 on MDC were determined in further experiments. MDC were stimulated with LPS and treated with 10 μM SB140. This concentration was chosen as SB140 affected the viability of peritoneal macrophages in doses higher than 10 μM. The treatment did not have a statistically significant effect on NO production (Figure 3A), IL-6 and TNF release (Figure 3B,C), or on the proportion of MDC expressing MHC class II molecules and CD86 (Figure 3D) or CD40 (Figure 3E). Therefore, SB140 did not influence the inflammatory properties of macrophages/dendritic cells.

### 2.3. SB140 Activates Nrf2 in Microglial Cells and MDC

**Figure 4:**
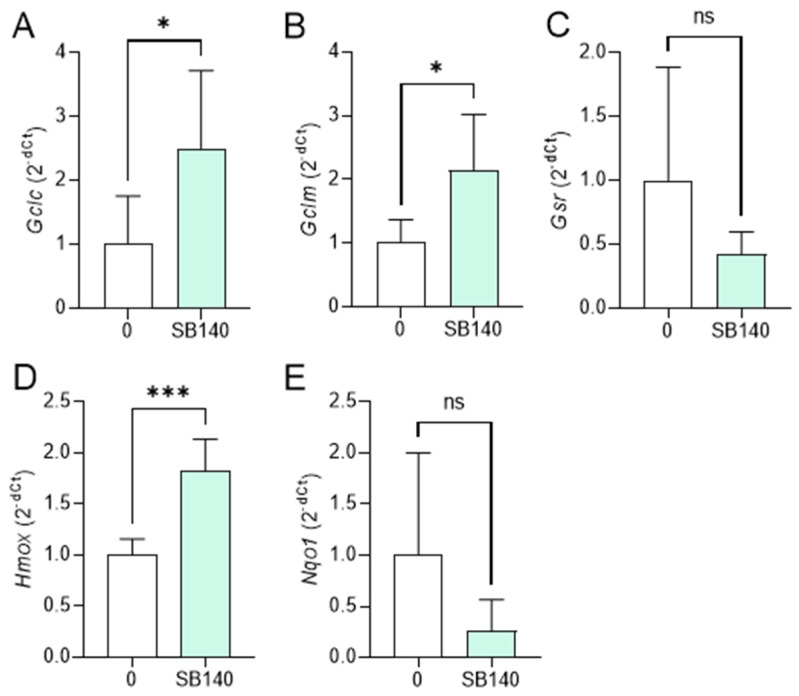
Effects of SB140 on nuclear factor-erythroid 2 related factor 2 (Nrf2)-regulated genes in microglial cells. BV2 cells were stimulated with IFN-γ (10 ng/mL) and LPS (10 ng/mL) in the absence (0) or presence of SB140 (40 μM) for 6 h. Subsequently, relative expression of *Gclc* (**A**), *Gclm* (**B**), *Gsr* (**C**), *Hmox* (**D**), and *Nqo1* (**E**) mRNA was determined by real-time RT-PCR. Data from five experiments are presented as mean + SD. * *p* < 0.05; *** *p* < 0.001; ns—no statistical difference; unpaired *t*-test.

**Figure 5:**
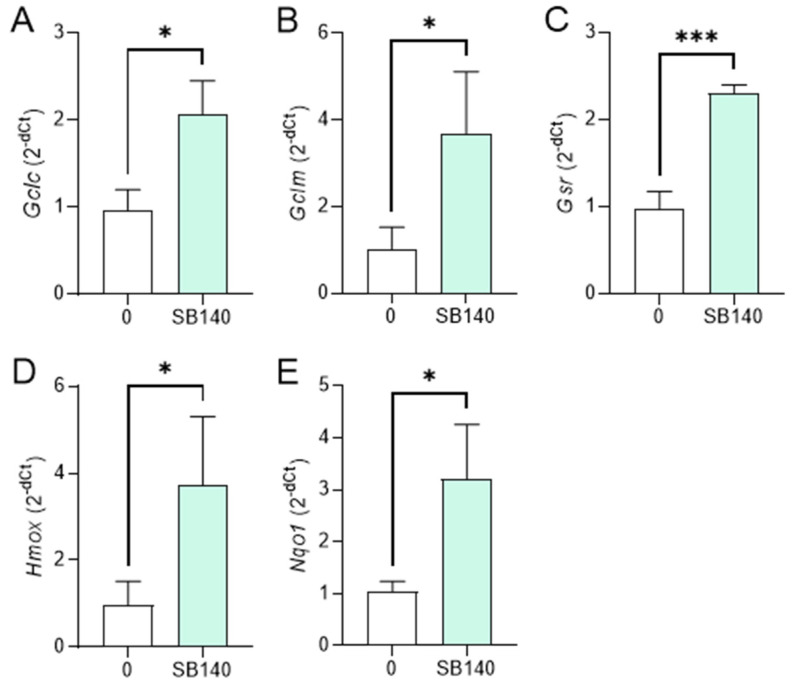
Effects of SB140 on Nrf2-regulated genes in MDC. MDC were stimulated with LPS (100 ng/mL) in the absence (0) or presence of SB140 (10 μM) for 6 h. Subsequently, relative expression of *Gclc* (**A**), *Gclm* (**B**), *Gsr* (**C**), *Hmox* (**D**), and *Nqo1* (**E**) mRNA was determined by real-time RT-PCR. Data from three experiments are presented as mean + SD. * *p* < 0.05; *** *p* < 0.001; unpaired *t*-test.

To explore the effect of SB140 on Nrf2 activation in microglial cells, BV2 cells were stimulated with IFN-γ+LPS and treated with 40 μM SB140 for 6 h, and the expression of mRNA for phase 2 response enzymes was determined. The treatment led to upregulation of mRNA expression for glutamate-cysteine ligase catalytic subunit (GCLC, gene *Gclc*), glutamate-cysteine ligase regulatory subunit (GCLM, gene *Gclm*), and heme oxygenase (HO-1, gene *Hmox*) (Figure 4A,B,D), but not for glutathione-disulfide reductase (GSR, gene *Gsr*), and NAD(P)H dehydrogenase quinone 1 (NQO1, gene *Nqo1*) (Figure 4C,E). To determine the effects of SB140 on Nrf2 activation in MDC, these cells were stimulated with LPS and treated with 10 μM SB140 for 6 h. The treatment led to upregulation of mRNA expression for GCLC, GCLM, GSR, HO-1, and NQO1 (Figure 5). Thus, SB140 led to Nrf2 activation both in microglial cells and MDC.

### 2.4. SB140 Exerts Immunomodulatory Effects on T Cells

**Figure 6:**
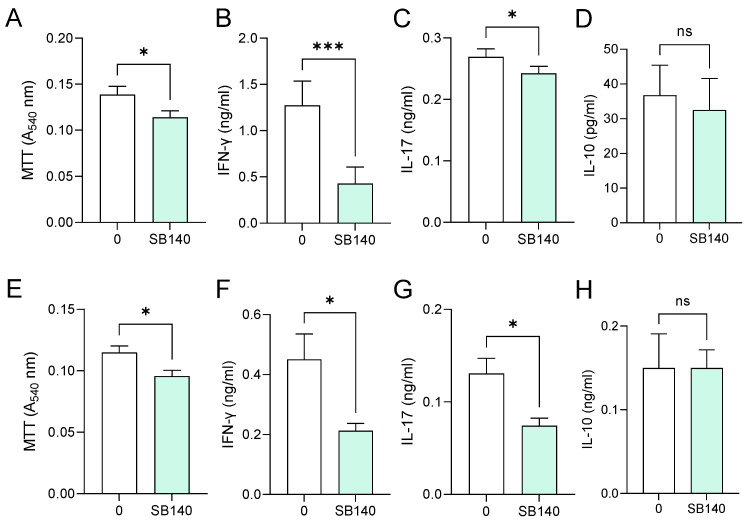
Effects of SB140 on spleen and lymph node cells. Spleen cells were obtained from MOG_35_–_55_-immunized mice (**A**–**D**). Lymph node cells were obtained from spinal cord homogenate (SCH)-immunized rats (**E**–**H**). Spleen cells were treated with MOG_35–55_, while lymph node cells were treated with myelin basic protein (MBP) in the absence (0) or presence of SB140 (10 μM) for 48 h. Subsequently, cell viability/proliferation was assessed by MTT assay (**A**,**E**), while levels of IFN-γ, IL-17, and IL-10 were determined in cell culture supernatants by ELISA (**B**–**D**,**F**–**H**). Data obtained from four (**A**–**D**) or three (**E**–**H**) samples are presented as mean + SD. * *p* < 0.05; *** *p* < 0.001; ns—no statistical difference; paired *t*-test.

**Figure 7:**
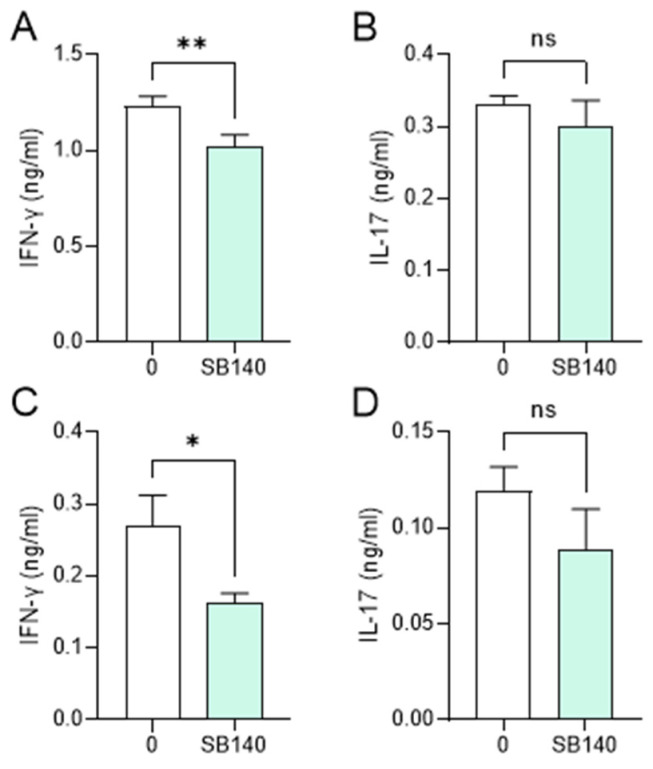
Effects of SB140 on T cells. Spleen cells were obtained from non-immunized mice. Spleen cells were treated with concanavalin A (ConA, 2 μg/mL) in the absence (0) or presence of SB140 (10 μM) for 24 h (**A**,**B**). CD4^+^ T cells sorted from spleen cells were treated with anti-CD3/anti-CD28 beads in the absence (0) or presence of SB140 (10 μM) for 48 h (**C**,**D**). Subsequently, levels of IFN-γ and IL-17 were determined in cell culture supernatants by ELISA. Data obtained from three (**A**,**B**) or four (**C**,**D**) samples are presented as mean + SD. * *p* < 0.05; ** *p* < 0.01; ns—no statistical difference; paired *t*-test.

Aiming to explore the effects of SB140 on encephalitogenic T cells, spleen cells were obtained from mice immunized with MOG_35–55_, while lymph node cells were obtained from rats immunized with spinal cord homogenate (SCH) (details provided in the Section 4). Spleen cells were stimulated with MOG_35–55_, while lymph node cells were stimulated with myelin basic protein (MBP) and treated with SB140. MTT test was performed to determine the effect of SB140 on cell viability/proliferation, while the levels of IFN-γ and IL-17, as a measure of T cell activity, were determined in cell culture supernatants. SB140 reduced the viability/proliferation of spleen and lymph node cells (Figure 6A,E) and downregulated the release of both IFN-γ and IL-17 from antigen-stimulated T cells (Figure 6B,C,F,G). Notably, IL-10 production was not affected at the same time (Figure 6D,H). Further, when spleen cells were stimulated with strong non-specific mitogen concanavalin A (ConA), SB140 was able to significantly reduce the release of IFN-γ, but not IL-17 (Figure 7A,B). Finally, to assess if SB140 acts directly on T cells, CD4^+^ T cells were sorted from spleen cells. SB140 efficiently reduced CD4^+^ T cell’s ability to produce IFN-γ, but not IL-17 (Figure 7C,D). Thus, SB140 efficiently restrained T cell inflammatory properties, but it exerted cytokine-specific effects.

## 3. Discussion

Here, we present that SB140 exerts anti-neuroinflammatory effects, as it reduces microglial production of NO, IL-6, and TNF and T cell production of IFN-γ and IL-17. The effect of SB140 on anti-inflammatory IL-10 in BV2 cells was limited, while the compound did not affect IL-10 production in antigen-stimulated lymphoid cells. Also, we show that the compound is a potent Nrf2 activator in microglial cells and MDC.

Activation of Nrf2 has been suggested as a viable strategy for the treatment of inflammatory neurodegenerative CNS diseases, such as multiple sclerosis, Parkinson’s disease, and Alzheimer’s disease [12,13]. Moreover, dimethyl fumarate, one of the disease-modifying drugs that is approved for the treatment of multiple sclerosis, is an Nrf2 activator [14]. Nrf2 activation was associated with a reduced ability of macrophages and microglia to generate inflammatory molecules [15,16]. Interestingly, in both studies, the effects of Nrf2 activation on inflammatory mediators were not achieved through ARE and phase 2 response genes but through transcriptional downregulation of inflammatory mediators and/or inhibition of NFκB. This may explain why, despite the efficacy of SB140 in upregulating the expression of phase 2 enzymes in both microglia and MDC, inflammatory mediators were affected only in the former in our study. Also, there is a possibility that SB140 affects immune cells’ ability to produce cytokines and other mediators independently of its effects on Nrf2, as it was previously shown that the generation of proinflammatory cytokines in T cells was not affected by changes in Nrf2 activity [17]. Thus, it will be important to address the effects of SB140 on NFκB and other intracellular signaling pathways relevant to the production of inflammatory mediators in microglia and macrophages/dendritic cells in future studies.

Microglia, a resident immune cell population in the CNS, plays a dominant role in the pathogenesis of various neurodegenerative and neuroinflammatory disorders, including Parkinson’s disease [9], Alzheimer’s disease [11], and multiple sclerosis [7]. Production of TNF, IL-6, and NO by microglia contributes to neurodegeneration and inflammation in these neurological diseases [7,9,11]. Thus, the ability of SB140 to reduce the production of TNF, IL-6, and NO in microglia implies that the compound might have beneficial effects in neurodegenerative and neuroinflammatory diseases. Furthermore, SB140 markedly increased the proportion of BV2 cells expressing CD206, a scavenging/phagocytosis marker that indicates an anti-inflammatory or immunomodulatory phenotype or behavior [7]. Intriguingly, SB140 also increased the proportion of BV2 cells expressing MHC class II molecules, thus potentially contributing to the potency of microglia to present antigens to CD4^+^ T cells. Still, the outcome of microglial antigen presentation to CD4^+^ T cells could result in either T cell activation or restriction [7]. Thus, the effect of SB140 on microglia-T cell interactions should be examined in future studies.

SB140 did not have an effect on the production of TNF, IL-6, and NO in macrophages/dendritic cells in our study. Also, it did not change the proportion of MDC expressing MHC class II molecules and costimulatory molecules, i.e., efficient antigen-presenting cells. Although macrophages contribute to the damage within the CNS in neurological disorders, they can also limit immune reactivity at the periphery. Namely, macrophages were shown to inhibit T cell proliferation through iNOS-mediated NO production [18], as well as through the release of TNF [19]. Also, IL-6 was shown to have direct immunomodulating effects on T cells [20], as well as to promote suppressive effects of myeloid cells on T cells [21]. Thus, the absence of the effect of SB140 on the production of TNF, IL-6, and NO in macrophages/dendritic cells might contribute to the limitation of T cell activation at the periphery and, therefore, could be positive in the context of inflammation prevention. Alternatively, macrophages producing these three biomolecules are considered M1 macrophages [22] that greatly contribute to the pathogenesis of inflammatory neurological disorders [6,8,10]. Thus, the lack of the influence of SB140 on their production in myeloid cells might limit its anti-inflammatory capacity. However, our study used just one of many possible in vitro systems for studying macrophages/dendritic cells, and different outcomes could be expected in alternative in vitro settings. Accordingly, SB140 has recently been shown to inhibit TNF production in peritoneal cells [23]. Therefore, it will be important to determine the effect of the compound on macrophages/dendritic cells, as well as on microglial cells, in vivo.

IFN-γ and IL-17 have a major role in the inflammatory response and consequent destruction of the CNS tissue in multiple sclerosis [24]. Indeed, their production by Th1 and Th17 cells was observed throughout the clinical course of the multiple sclerosis animal model, experimental autoimmune encephalomyelitis (EAE) [25]. Hence, the ability of SB140 to modulate the production of these cytokines in T cells implies that the compound has the potential to act against CNS autoimmunity. Along the same line, SB140 did not affect the ability of immune cells to produce anti-inflammatory IL-10, thus further substantiating the assumption that it might be effective against CNS autoimmunity. Interestingly, while SB140 was able to reduce the production of both cytokines in antigen-specific systems, it inhibited IFN-γ, but not IL-17, in polyclonally activated T cells, as well as in purified CD4^+^ T cells. These results imply that the compound acts specifically on some intracellular pathways that are important for IFN-γ synthesis but not for the synthesis of IL-17 in T cells. Also, it seems that SB140 acts on IFN-γ, but not IL-17, production in CD4^+^ T cells directly. This divergence in the inhibitory effect of SB140 might be important for its potential anti-encephalitogenic effects, as it was previously shown that T cells producing IFN-γ and IL-17 dominate in spinal cord and brain autoimmunity, respectively [26]. Thus, it will be important to apply SB140 in vivo in EAE to determine its effects on CNS autoimmunity.

## 4. Materials and Methods

### 4.1. SB140

SB140 was synthesized as previously described (compound **25** in [1]). SB140 was dissolved in dimethyl sulfoxide at a 20 mM concentration and further diluted in cell culture media.

### 4.2. Cells and Cell Cultures

BV2 cells (a kind gift of Dr. Alba Minelli, Università degli Studi di Perugia, Perugia, Italy) were grown in RPMI-1640 (Capricorn Scientific, Ebsdorfergrund, Germany) supplemented with 10% fetal bovine serum (FBS, Capricorn Scientific) in 24-well plates (3 × 10^5^/mL). BV2 cells were stimulated with murine IFN-γ (10 ng/mL, Peprotech, London, UK) and LPS (10 ng/mL, Sigma-Aldrich, Saint Louis, MO, USA) for 24 h.

Dark Agouti (DA) rats and C57BL/6 mice were bred and maintained in the animal facility of the Institute for Biological Research “Siniša Stanković” and the procedures were performed under permissions granted by the Veterinary Administration, Ministry of Agriculture, Forestry and Water Management, Republic of Serbia (No 323-07-10742/2022-05, and 323-07-11565/2022-05). C57BL/6 mice were immunized with MOG_35–55_ (SB-PEPTIDE, Saint-Egrève, France) mixed with complete Freund’s adjuvant (CFA). MOG_35–55_ was at a concentration of 1.5 mg/mL. CFA was obtained by supplementing incomplete Freund’s adjuvant with *M. tuberculosis* (10 mg/mL, both from BD Biosciences, Franklin Lakes, NJ, USA). MOG_35–55_ and CFA were mixed at a 1:1 volume ratio. Mice were injected with 200 μL of MOG_35–55_ + CFA by administering two 100 µL injections into each lateral side of the lower back. Mice were injected intraperitoneally with Pertussis toxin (250 ng/mouse, Biotechne, Minneapolis, MN, USA) on the day of the immunization and 48 h later. DA rats were immunized with syngeneic rat SCH in phosphate-buffered saline (PBS, 50% *w*/*v*), as previously described [27]. Spleens were obtained from mice on day 29 post-immunization or from healthy, non-immunized mice. Clinical scores of the immunized mice were 1.5–2.5. Cells of the lymph nodes draining the site of immunization—popliteal lymph node cells were isolated from rats on day 6 post-immunization. Rats did not have overt clinical signs at the time. Spleens and lymph nodes were mechanically disrupted in PBS supplemented with 3% FBS, using a syringe plunger and 70 μm nylon mesh strainer (Corning, Glendale, AZ, USA) within a petri dish (Sarstedt, Nümbrecht, Germany) to obtain single-cell suspension. In addition, red blood cell lysis was performed on spleen single-cell suspensions by an ammonium chloride-based buffer. CD4^+^ T cells were obtained from spleen cells by sorting on BD FACSAria™ III Cell Sorter (BD Biosciences), using FITC-conjugated anti-CD4 antibody (RM4-5, Thermo Fisher Scientific, Waltham, MA, USA) for labeling. Both spleen and lymph node cells were seeded in 24-well plates (5 × 10^6^/mL). Spleen cells were stimulated with MOG_35–55_ (10 µg/mL) in RPMI-1640 supplemented with 2% mouse serum for 48 h or with 2 µg/mL ConA (Pharmacia Fine Chemicals, Uppsala, Sweden) in RPMI-1640 supplemented with 5% FBS for 24 h, while lymph node cells were stimulated with guinea pig MBP (10 µg/mL, kind gift of prof Alexander Flügel, IMSF, University of Gottingen, Germany) in RPMI-1640 supplemented with 2% rat serum for 48 h. CD4^+^ T cells were seeded in U-bottom 96-well plates (8 × 10^4^/mL). They were stimulated with Dynabeads™ Mouse T-Activator CD3/CD28 for T-Cell Expansion and Activation as suggested by the manufacturer (Thermo Fisher Scientific), in RPMI-1640 supplemented with 5% FBS for 48 h. Spleen cells and lymph node cells were obtained from individual immunized animals and used for both SB140-treated and non-treated cultures. Thus, the effects of SB140 were determined through a paired comparison of cultures that arose from the same animal in all experiments.

MDC were obtained from non-immunized C57BL/6 mice, as previously described [28]. The cells were stimulated with 100 ng/mL of LPS in RPMI-1640 supplemented with 10% FBS for 24 h.

### 4.3. Viability Assays

The viability of BV2 cells was assessed by crystal violet (CV) assay, and the viability/proliferation of splenocytes and lymph node cells was assessed using the MTT test, as described previously [29]. The absorbance of dissolved dyes, corresponding to the number of viable cells, was measured in triplicates at 540 nm with a correction at 670 nm using an automated microplate reader Synergy H1 (Agilent Technologies, Santa Clara, CA, USA).

### 4.4. Detection of NO Release

Nitrite accumulation, a measure of NO release, was determined in BV2 cells and MDC culture supernatants using the Griess reaction, as previously described [29]. The absorbance at 540 nm was determined with a microplate reader (Synergy H1) and compared to a standard curve for NaNO_2_.

### 4.5. ELISA

Cytokine concentrations in cell-free culture supernatants were determined by the sandwich ELISA method using MaxiSorp plates (Nunc, Rochild, Denmark). Anti-cytokine paired antibodies were used according to the manufacturers’ instructions (mouse/rat IL-17, mouse IFN-γ, rat IFN-γ, mouse IL-6, rat IL-10—Thermo Fisher Scientific; mouse TNF—Abcam, Cambridge, UK, mouse IL-10—R&D Systems, Minneapolis, MN, USA). The antibodies were as follows: anti-mouse/rat IL-17A purified rat monoclonal (eBio17CK15A5), anti-mouse/rat IL-17A biotinylated rat monoclonal (eBio17B7), anti-mouse/rat IFN-γ purified mouse monoclonal (XMG1.2), anti-human/mouse IFN-γ biotinylated rat monoclonal (R4-6A2), anti-rat IFN-γ purified mouse monoclonal (DB1), anti-rat IFN-γ biotinylated rabbit polyclonal, anti-mouse IL-6 purified mouse monoclonal (MP5-20F3), anti-mouse IL-6 biotinylated mouse monoclonal (MP5-32C11), anti-rat IL-10 capture and detection antibodies (catalog No 88-50629), anti-mouse TNF purified rabbit monoclonal (EPR16803-2), anti-mouse TNF biotinylated rabbit monoclonal (EPR16803-84), anti-mouse IL-10 purified mouse monoclonal (JES516E3), and anti-mouse IL-10 biotinylated mouse monoclonal (JES52A5). The absorbance was recorded at 450 nm with a correction at 670 nm using a microplate reader Synergy H1. Samples were analyzed in duplicates, and the results were calculated using standard curves based on known concentrations of the recombinant rat IFN-γ and rat IL-17 (Peprotech, London, UK), mouse IL-17 (R&D Systems), mouse IL-6 (Thermo Fisher Scientific), and mouse TNF (Abcam). For all ELISA tests, the lower limit of detection was 30 pg/mL, while the upper limit of detection was 10 ng/mL.

### 4.6. Flow Cytometry

The following antibodies (all from Thermo Fisher Scientific, except anti-CD11b, which was from Biolegend, San Diego, CA, USA) were used for cell staining: FITC-conjugate anti-mouse F4/80 (BM8), PE-conjugated anti-mouse/rat MHC II (14-4-4S), APC-conjugated anti-mouse MHC II (M5/114.15.2), PE-conjugated anti-mouse CD206 (MR6F3), PE-Cy5-conjugated anti-mouse CD86 (GL1), eFluor™ 450-conjugated anti mouse/rat CD40 (HM40-3), biotin-conjugated anti-mouse CD11b (M1/70) coupled with streptavidin APCCy7. Adequate isotype control antibodies were used where necessary to set the gates for cell marker positivity. Typically, the proportion of isotype control antibody-stained cells was <1%. The acquisition of the samples was performed using a CytoFLEX flow cytometer (Beckman Coulter, Indianapolis, IN, USA) and analyzed using CytExpert software v2.4.0.28 (Beckman Coulter). Results of cytofluorimetry are presented as percentages of cells.

### 4.7. Reverse Transcription−Real-Time Polymerase Chain Reaction

Total RNA was isolated from cells using a mi-Total RNA Isolation Kit (Metabion, Martinsried, Germany). Reverse transcription was performed using random hexamer primers and MMLV (Moloney Murine Leukemia Virus), according to the manufacturer’s instructions (Fermentas, Vilnius, Lithuania). Prepared cDNAs were amplified by using Maxima SYBR Green/ROX qPCR Master Mix (Fermentas, Vilnius, Lithuania) according to the recommendations of the manufacturer in the QuantStudio 3 (Applied Biosystems, Foster City, CA, USA). Thermocycler conditions comprised an initial step at 50 °C for 5 min, followed by a step at 95 °C for 10 min, and a subsequent two-step PCR program at 95 °C for 15 s and 60 °C for 60 s for 40 cycles. The PCR primers (Metabion) were as follows: β-actin: 5′-CCA GCG CAG CGA TAT CG-3′; 5′-GCT TCT TTG CAG CTC CTT CGT-3′; *Gclc*: 5′-CAT CTA CCA CGC AGT CAA GG-3′; 5′-TCA TGA TCG AAG GAC ACC AA-3′; *Gclm*: 5′-ATG CTC CGT CCT TGG AGT T-3′; 5′-GCT GCT CCA ACT GTG TCT TG-3′; *Gsr*: 5′-GCT CCA TCT CAG TCC GTT CTT-3′; 5′-GAT TCC TGC AGG CCT TAA CC-3′; *Hmox1*: 5′-TAA GCT GGT GAT GGC TTC CT-3′; 5′-TCT GCT TGT TGC GCT CTA TC-3′; *Nqo1*: 5′-GAT CCT GGA AGG ATG GAA GA-3′; 5′-TCT GGT TGT CAG CTG GAA TG-3′. PCR product accumulation was detected in real-time, and for the analysis of the results, QuantStudio^TM^ Design&Analysis Software v1.4.3 (Applied Biosystems, Foster City, CA, USA) was used. Relative mRNA expression is presented as 2^−dCt^, where dCt is the difference between Ct values of a gene of interest and the endogenous control (β-actin).

### 4.8. Statistics

The significance of the differences between the groups was determined by repeated measures of one-way ANOVA followed by Dunnett’s multiple comparisons test or with *t*-test using GraphPad Prism version 9.00 for Windows (GraphPad Software, La Jolla, CA, USA), as reported in the figure legends.

## 5. Conclusions

Our results clearly show that SB140 exerts potent immunomodulatory effects on microglial cells and encephalitogenic T cells in vitro. Its profound effect on Nrf2 activation encourages further studies on the pharmacological modulation of various diseases characterized by impaired Nrf2 activation. It has to be noted that the effects of SB140 on Nrf2 activation in this study were determined only on the mRNA level of downstream molecules. Although the upregulation of Nrf2 target genes is often considered as the marker of Nrf2-ARE pathway activation [14,30], it will be important to explore whether these effects also reflect a functional activation at the protein level. Thus, our results stimulate further studies on the anti-neuroinflammatory effects of SB140. Such studies should be performed in vivo in animal models of multiple sclerosis and other inflammatory neurological disorders.

## Data Availability

Data is contained within the article. Additional information is available on request from the corresponding author.
